# Reproducibility between three-dimensional turbo spin-echo and two-dimensional dual inversion recovery turbo spin-echo for coronary vessel wall imaging in Kawasaki disease

**DOI:** 10.1038/s41598-022-10951-0

**Published:** 2022-04-27

**Authors:** Koji Matsumoto, Hajime Yokota, Takafumi Yoda, Ryota Ebata, Hiroki Mukai, Yoshitada Masuda, Takashi Uno

**Affiliations:** 1 Department of Radiology, Chiba University Hospital, 1-8-1, Inohana, Chuo-ku, Chiba, Japan; 2 Diagnostic Radiology and Radiation Oncology, Graduate School of Medicine, Chiba University, Chiba, Japan; 3 Department of Pediatrics, Graduate School of Medicine, Chiba University, Chiba, Japan

**Keywords:** Cardiology, Magnetic resonance imaging, Three-dimensional imaging

## Abstract

Magnetic resonance vessel wall imaging is desirable for evaluating Kawasaki disease (KD)-associated coronary arterial lesions. To evaluate the reproducibility of three-dimensional turbo spin-echo (3D-TSE) and two-dimensional dual inversion-recovery turbo spin-echo (2D-DIR-TSE) for coronary vessel wall imaging in KD. Ten patients were prospectively enrolled. Coronary vessel wall imaging with axial-slice orientation 3D-TSE and 2D-DIR-TSE were acquired for cross-sectional images in aneurysmal and normal regions. Lumen area (LA), wall area (WA), and normalized wall index (NWI) of cross-sectional images were measured in both regions. Reproducibility between 3D-TSE and 2D-DIR-TSE was evaluated via intraclass correlation coefficients (ICCs) and Bland–Altman plots. 48 points (aneurysmal, 27; normal, 21) were evaluated. There were high ICCs between 3D-TSE and 2D-DIR-TSE in LA (0.95) and WA (0.95). In aneurysmal regions, 95% limits of agreement were LA, WA, and NWI of − 29.9 to 30.4 mm^2^, − 18.8 to 15.0 mm^2^, and − 0.22 to 0.20, respectively. In normal regions, the 95% limits of agreement were LA, WA, and NWI of − 4.44 to 4.38 mm^2^, − 3.51 to 4.30 mm^2^, and − 0.14 to 0.16, respectively. No fixed and proportional biases between 3D-TSE and 2D-DIR-TSE images in aneurysmal and normal regions were noted. 3D-TSE was reproducible with conventional 2D-DIR-TSE for coronary vessel wall assessment on KD.

## Introduction

Kawasaki disease (KD) is an acute febrile illness of unknown etiology that usually occurs in children below 5 years of age and is a systemic vasculitis syndrome^1^. Coronary arteritis is caused in the majority of patients with acute KD^2^, often resulting in coronary artery aneurysms. Aneurysm formation in KD is considered secondary to the destruction of the internal elastic membrane because of severe coronary angiitis^3^. Coronary artery aneurysms can rupture, thrombose, and lead to the development of stenotic lesions. Serious coronary complications reportedly cause ischemic heart disease in children and are now the most common cause of pediatric coronary disease worldwide^4^.

Coronary magnetic resonance angiography (MRA) using three-dimensional (3D) balanced steady-state free precession imaging, a minimally invasive procedure, is widely used for evaluating coronary arterial lesions in KD^5^. However, MRA does not allow the sufficient evaluation of coronary artery wall changes, such as thrombi and intimal proliferation. Intravascular ultrasound (IVUS) or optical coherence tomography (OCT) is the standard approach for coronary artery wall assessments^6,7^. However, IVUS and OCT are invasive techniques with non-negligible risks and are difficult to perform routinely. Transthoracic echocardiography (TTE)^8^ can be generally used to evaluate the coronary vessel wall up to early childhood. Nonetheless, it is difficult to conduct TTE after adolescence. This necessitates a non-invasive method that can be examined frequently from the acute to the follow-up phase. Therefore, magnetic resonance vessel wall imaging is desirable for evaluating KD-associated coronary arterial lesions^9^.

Previous reports have used two-dimensional dual inversion-recovery turbo spin-echo (2D-DIR-TSE)^9,10^ and three-dimensional turbo spin-echo (3D-TSE)^11^ imaging for pediatric coronary vessel wall imaging. 2D-DIR-TSE imaging is often challenging when setting the field-of-view (FOV) in clinical cases. This can be attributed to the occurrence of coronary artery aneurysms in multiples and at bifurcations. This necessitates the use of 3D-TSE imaging that facilitates extensive coverage. Any section or direction can be reconstructed and visualized using wide-FOV-imaging-derived 3D volume data. In contrast, 2D-DIR-TSE imaging has the advantage of obtaining a cross-sectional image with high in-plane resolution in a short scan time. The disadvantages of 3D-TSE imaging include motion artifacts because of the long scan time. In addition, the flow direction, with respect to the slice encoding direction, affects the suppression of the blood signal. The problems of 2D-DIR-TSE imaging have not been completely evaluated previously. Thus, it is necessary to compare the reproducibility and effectiveness of 3D-TSE and 2D-DIR-TSE imaging. Therefore, we aimed to perform qualitative and quantitative evaluations of cross-sectional vessel wall images collected by 3D-TSE and 2D-DIR-TSE imaging in KD and compare their reproducibility.

## Methods

### Subjects

Approval was provided by Chiba University Hospital (approval number, 3901; Registered 14 December 2020), and written informed consent was obtained from all patients or their families. This study was performed in accordance with relevant guidelines and regulations, including the Declaration of Helsinki. We prospectively enrolled ten patients (five each, female and male; mean age ± standard deviation [SD], 12.9 ± 7.2 years; range, 5–25 years) who underwent cardiac magnetic resonance imaging (MRI) between September 2015 and October 2019. These patients were undergoing follow-up care for a dilated lesion of the coronary artery during the acute phase of KD or coronary arterial lesions, such as aneurysms and stenosis resulting from KD.

TTE and invasive coronary angiography (ICA) revealed aneurysms at 15 sites in 10 patients (right coronary artery [RCA], five; left coronary artery [LCA], ten). Of the ten patients, five aged < 9 years underwent MRI examination under sedation. To achieve mild patient sedation during the MRI examination, they were administered sodium trichloroethylene phosphate syrup (0.7 to 0.8 mL/kg). However, they were administered thiopental sodium (2–3 mg/kg) or midazolam (0.2 mg/kg) via IV infusion if the syrup was ineffective to achieve unconscious sedation for the duration of imaging.

### Data acquisition

**Figure 1:**
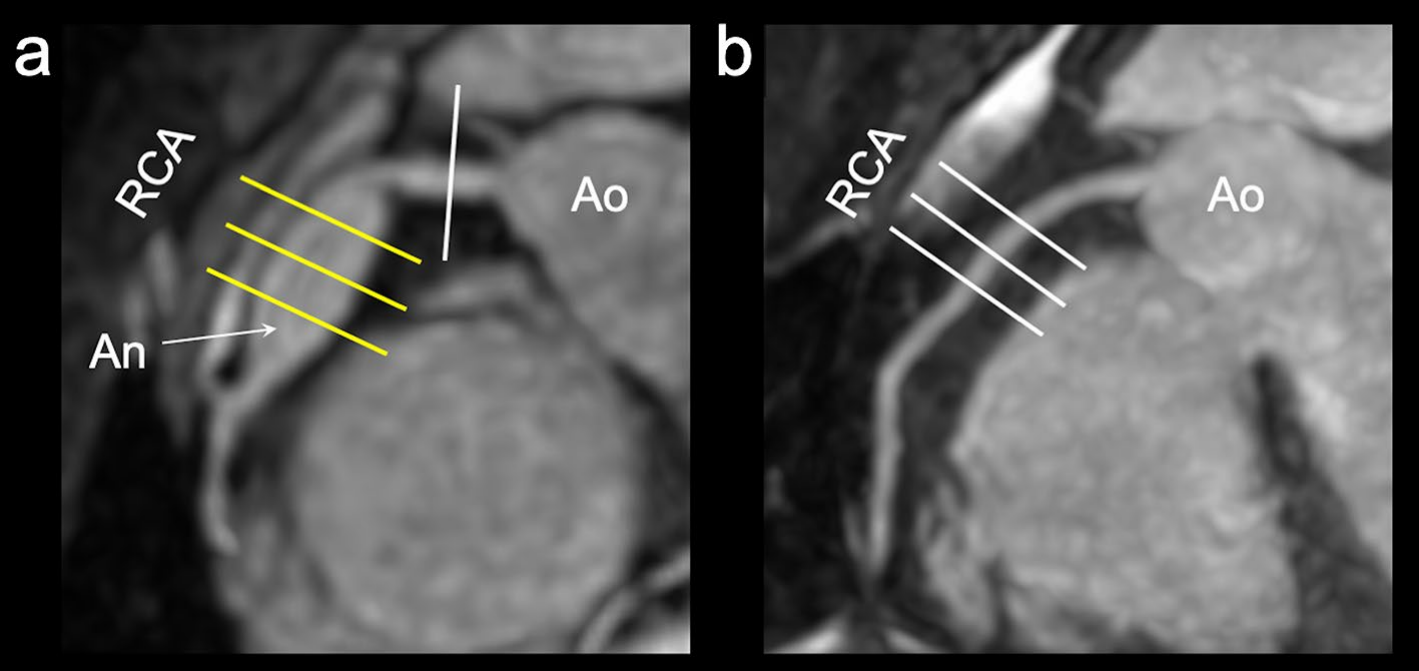
Slice setting. The white lines indicate normal regions, and the yellow lines indicate aneurysmal regions. The magnetic resonance angiography (MRA)-collapsed partial maximum intensity projection (MIP) image demonstrates the right coronary artery (RCA) in case 7 (**a**). For aneurysms, the basic settings were three cross-sections: center, proximal, and distal portions (**a**). The MRA-collapsed partial MIP image demonstrates the RCA in case 8 (**b**). In the follow-up examination of aneurysm regression, the previous aneurysm regression was regarded as a normal part, and cross-sectional images were continuously obtained (**b**) (*An* aneurysm, *Ao* aorta).

All MRI studies were performed on a 1.5-Tesla MRI unit (Intera Achieva Nova Dual Release 5, Philips Healthcare, Best, The Netherlands) using 32-elements ds Anterior and ds Posterior coils. First, we used a high-resolution 2D cine scan with an axial slice orientation to identify the precise period of minimal cardiac motion in each patient^12^. Subsequently, we performed MRA using 3D balanced steady-state free precession (3D balanced turbo field-echo) with an axial slice orientation to confirm the target part and image the entire heart. Next, 3D-TSE imaging was performed with axial slice orientation in the proximal regions of the coronary artery. Moreover, we acquired cross-sectional images of the aneurysm from multiple cross-sections (e.g., center, proximal, and distal portions) and images of normally appearing juxta-proximal and juxta-distal points of the aneurysms with 2D-DIR-TSE imaging. Figure 1 depicts an example of a slice setting.

3D-TSE images were obtained with the following parameters: (i) a free-breathing navigator-gated and cardiac-triggered 3D turbo spin-echo with a repetition time (TR) equivalent to the duration of one heartbeat (698 ms to 896 ms); (ii) an echo time (TE) of 36 ms to 62 ms; (iii) a refocusing flip angle of 50°; (iv) a turbo factor of 14 to 22; (v) an acquired spatial resolution of 1.17 × 1.25 × 1.40 mm (reconstruction, 0.55 × 0.55 × 0.70 mm); (vi) a slice number of 60; and (vii) flow-sensitizing to suppress the blood signal^13^. The acquisition window was set to 40–70 ms, depending on the period of minimum cardiac motion for each patient. We used a 50° refocusing flip angle to increase the saturation. Flowing spin in fast spin-echo imaging arises from two primary mechanisms as follows: intravoxel dephasing from isochromats with different velocities and mixing among pathways involving different combinations of spin echoes and stimulated echoes^14^. The use of low refocusing flip angles further promotes the phase dispersion of flowing spins^14^. Furthermore, it slows the decay of the signal from stationary tissues, thereby allowing longer echo trains or a narrower echo spacing^15^. In addition, we applied a spectral inversion recovery (SPIR) pulse to suppress the epicardial fat signals.

In contrast, 2D-DIR-TSE images were obtained with the following parameters: (i) a free-breathing navigator-gated and cardiac-triggered 2D turbo spin-echo with a TR equivalent to the duration of one heartbeat (698 ms to 896 ms); (ii) a TE of 20 ms^10^; (iii) a turbo factor of 9; (iv) a resolution of 0.98 × 0.96 × 4.00 mm (acquired spatial reconstruction, 0.49 × 0.49 × 4.00 mm); and (v) an SPIR and acquisition window of 45 ms.

All sequences were equipped with a right hemi-diaphragmatic prospective real-time navigator for suppressing the respiratory motion artifact in patients with free breathing^16^. Data were accepted if the lung-liver interface was within the end-expiratory gating window (1–4 mm). However, they were rejected and reacquired during the subsequent inter-beat interval.

### Postprocessing

Coronary vessel walls were evaluated separately in the aneurysmal and normal regions diagnosed via TTE or ICA. A coronary aneurysm was diagnosed if the internal lumen diameter was > 4.0 mm or on observing distension of a part of a coronary vessel of up to one and a half times the diameter of an adjacent normal segment^17^.

To evaluate the coronary vessel wall and lumen size, we reformatted the 3D-TSE dataset for same-angle cross-sectional images as 2D-DIR-TSE on a commercial workstation (OsiriX Dicom viewer Version 12.0, Pixmeo SARL, Switzerland, https://www.osirix-viewer.com/). To minimize digitization errors, the cross-sectional images were reconstructed using 768 matrices. In the coronary vessel wall images, we set the window level and width at the level to diminish the intraventricular blood signal.

### Visual grading

**Figure 2:**
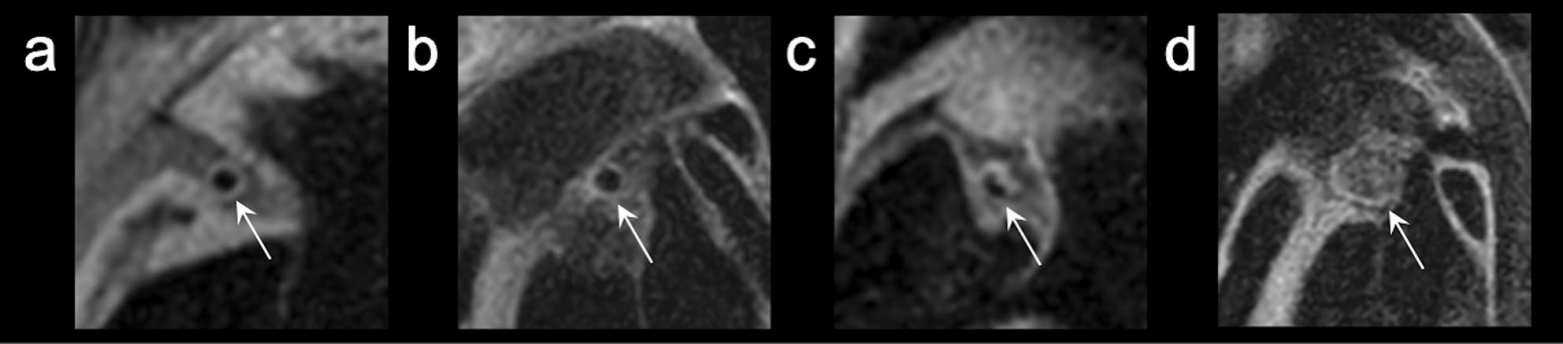
Image quality assessment of lumen boundary visualization quality (arrows in (**a–d**), grades 4–1). Lumen boundary visibility was assessed on a four-point scale for each coronary artery segment. Grade 4, excellent (no flow artifacts, lumen boundary was clearly shown); grade 3, good (few flow artifacts, lumen boundary was not obscured); grade 2, fair (moderate flow artifacts, a part of lumen boundary was unclear); grade1, bad (strong flow artifacts, lumen boundary was not visible, not diagnostic).

**Figure 3:**
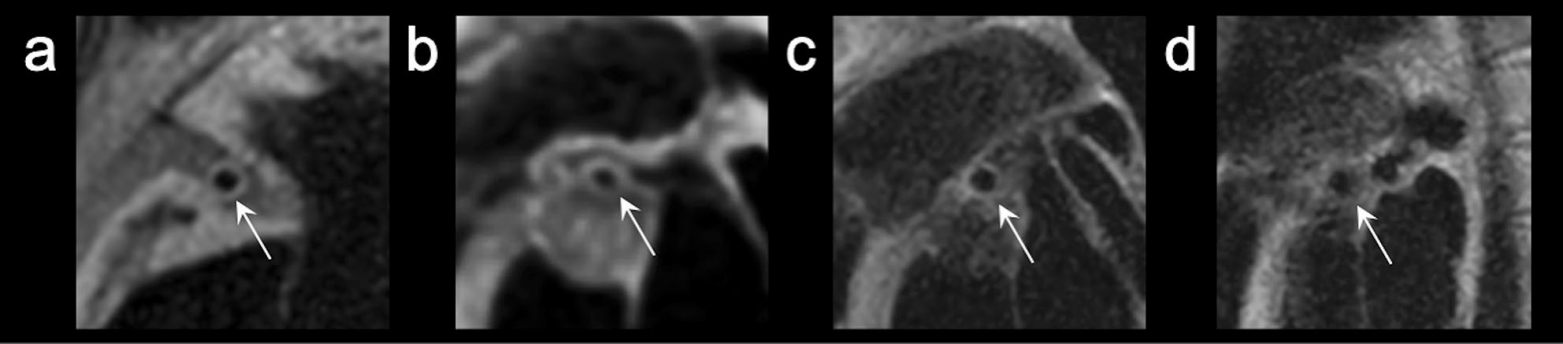
Image quality assessment of outer wall boundary visualization quality (arrows in (**a–d**), grades 4–1). Lumen boundary visibility was assessed on a four-point scale for each coronary artery segment. Grade 4, excellent (entire vessel wall was clearly visible); grade 3, good (vessel wall was visible, but a portion was unclear); grade 2, fair (vessel wall was unclear, but 2/3 circumference was visible); grade1, bad (more than 1/3 of the vessel wall was not visible).

Two board-certified radiologists with 15 and 13 years of experience independently evaluated all coronary vessel wall images. The quality of the lumen boundary and outer wall boundary of each cross-sectional image was visually graded using a four-point grade (4, excellent; 3, good; 2, fair; and 1, bad). Figures 2 and 3 depict the four-point grade criteria^18^. Any discrepancies between the two readers were resolved by consensus.

### Measuring the lumen area, wall area, and normalized wall index

The lumen and outer wall contours of each cross-sectional image were manually traced using the OsiriX Dicom viewer. Based on these contours, the software program automatically calculated the lumen area (LA) and total vessel area (TVA) for each analyzed image. The wall area (WA) was measured by subtracting the LA from the TVA. In addition, to adjust for differences in the vessel size within each patient, we calculated a normalized wall index (NWI)^19^. The NWI was calculated as follows:

$$ {\text{NWI}} = {\text{WA}}/{\text{TVA}}. $$

**Figure 4:**
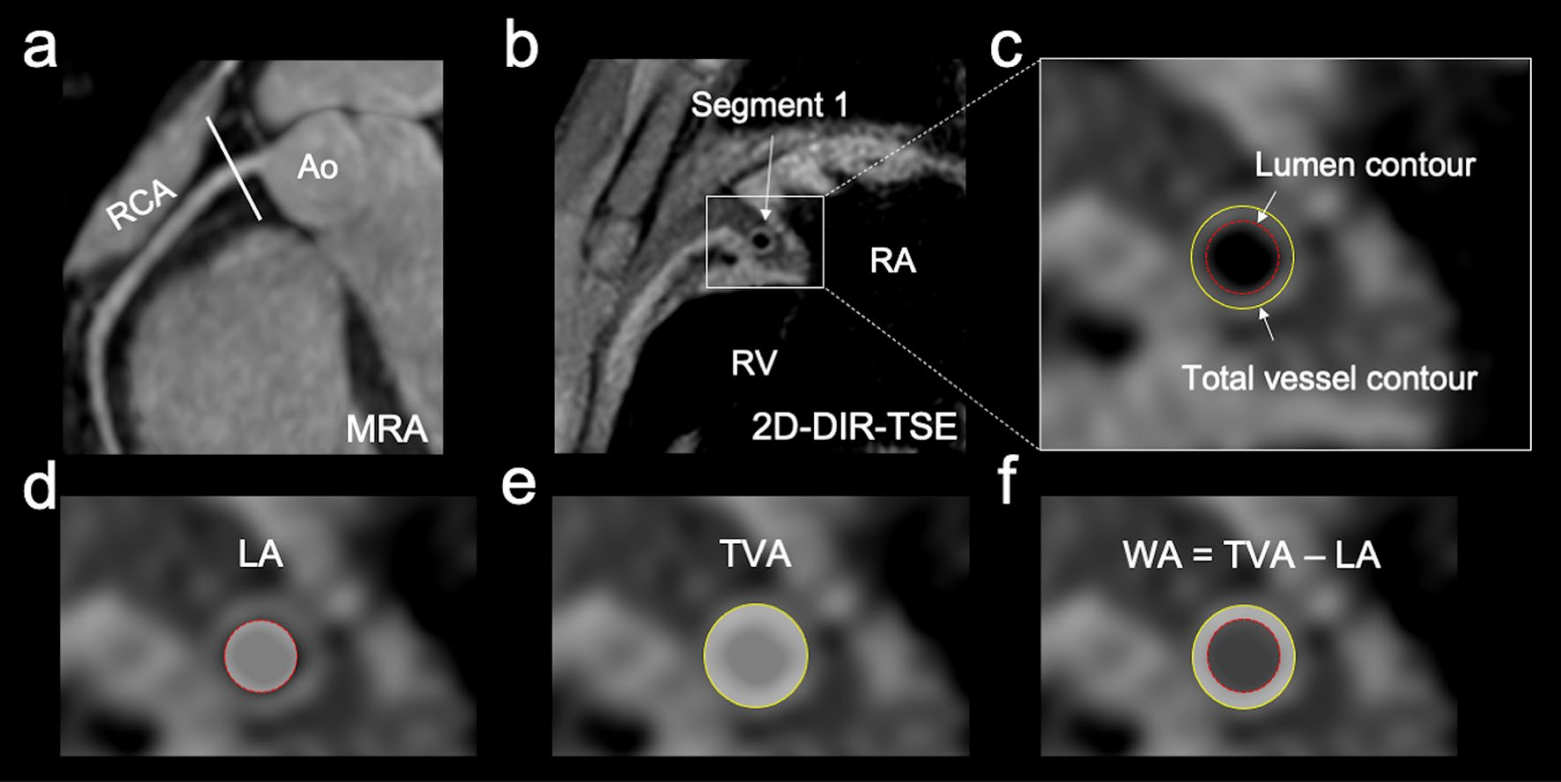
Regions of interest segmentation. The MRA-collapsed partial MIP image demonstrates RCA segments 1 in a 19-year-old boy (case 8) (**a**). At 6 months of age, he experienced a coronary artery aneurysm of up to 5.9 mm in RCA segments 1. It is currently involuted and divided into normal regions. The white line depicts the slice position in the two-dimensional dual inversion-recovery turbo spin-echo (2D-DIR-TSE) cross-sectional image (**b**). Image (**c**) is an enlarged image of the measurement location of (**b**). The lumen of the coronary artery (red dotted line) and the boundaries of the wall (solid yellow line) have been manually contoured (**c**). Subsequently, the lumen area (LA), the total vessel area (TVA), and the wall area (WA) have been measured (**d–f**) (*Ao* aorta, *RA* right atrium, *RV* right ventricle).

Figure 4 depicts the quantification of the coronary vessel wall characteristics. Each area measurement was performed in all cases with a visual grade between 2 and 4.

### Inter- and intra-reader reproducibility

To determine the intra-reader reproducibility, an experienced reader (A, 20 years of image analysis experience) analyzed all cross-sectional images. The recall bias was reduced to a minimum by using a time frame of approximately 180 days between the first and second image analyses. In contrast, to determine the inter-reader reproducibility, another reader (B, 4 years of image analysis experience) independently analyzed similar images as A (Fig. 2)^19^. Both readers were blinded to the patient’s characteristics and results. The blinding was ensured by replacing each patient’s image identifier with a pre-assigned unique study number.

### Statistical analyses

All statistical analyses were performed using R version 3.6.3 (The R Foundation for Statistical Computing, Vienna, Austria). To compare the visual grading of 3D-TSE and 2D-DIR-TSE images, we performed Wilcoxon signed-rank tests. *P*-values < 0.05 were considered statistically significant. To compare the reproducibility between 3D-TSE and 2D-DIR-TSE images, we used intraclass correlation coefficients (ICCs) and Bland–Altman plots. Fixed and proportional biases were defined as significant with *P*-values < 0.05, using a paired t-test and linear regression.

ICCs were used to evaluate the inter-reader and intra-reader reproducibility.

## Results

We evaluated a total of 48 coronary artery points (aneurysmal regions, 27; normal regions, 21). Table 1 summarizes the American Heart Association segments and the number of points evaluated from all patients with KD. Lumen diameter measured by TTE or ICA performed within 3 months is also shown. No thromboses were identified via TTE or ICA. No adverse clinical events occurred during the period between cardiac MRI and TTE or ICA. The heart rates during MRI ranged between 63 and 81 beats per minute (BPM) (mean ± SD: 72.1 ± 6.6 BPM). The actual scan time for each case was approximately 9–11 min and 1–2 min for 3D-TSE imaging and 2D-DIR-TSE imaging, respectively. All imaging was performed during mid-diastole, a quiescent period in the cardiac cycle.

**Table 1:** The number of points evaluated in 10 patients with Kawasaki disease.

Patients (age [years] and gender)	AHA segment (s)	Aneurysm		Normal	
		Number of points	TTE/ICA [mm]	Number of points	TTE/ICA [mm]
Case 1 (8F)	6	4	8.3	1	N/A
Case 2 (8M)	5	N/A	N/A	3	3.0
	6	N/A	N/A	2	2.0
Case 3 (6F)	1	3	3.7–4.5	2	2.3–2.7
Case 4 (16F)	5	5	13.0	N/A	N/A
Case 5 (25M)	1	N/A	N/A	3	N/A
	5	N/A	N/A	3	N/A
Case 6 (11F)	6	3	7.9	1	3.3
Case 7 (6M)	1	N/A	N/A	1	N/A
	2	3	N/A	N/A	N/A
Case 8 (19M)	1	N/A	N/A	3	N/A
	5	N/A	N/A	1	N/A
	6	N/A	N/A	1	N/A
Case 9 (5M)	2	3	9.3	N/A	N/A
	6	3	16.5	N/A	N/A
Case 10 (20F)	5	3	N/A	N/A	N/A
Total		27		21	

*AHA* American Heart Association, *TTE* transthoracic echocardiography, *ICA* invasive coronary angiography, *N/A* not applicable.

Table 2 summarizes the visual grading of the 3D-TSE and 2D-DIR-TSE images. Grade 3 or higher points in the aneurysmal regions were 25.9% and 18.5% at the lumen boundary, and 22.2% and 18.5% at the outer wall boundary for 3D-TSE and 2D-DIR-TSE images, respectively. Conversely, grade 3 or higher points in the normal regions were 95.2% for both 3D-TSE and 2D-DIR-TSE images at the lumen boundary, and 42.9% and 52.4% for 3D-TSE and 2D-DIR-TSE images, respectively, at the outer wall boundary. There were no significant differences between the 3D-TSE and 2D-DIR-TSE images in the lumen and outer wall boundaries. At the lumen boundary in the aneurysm regions, the 3D-TSE images had a higher visual grade than the 2D-DIR-TSE images. However, this difference was statistically insignificant (*P* = 0.073). At the outer wall boundary in the normal regions, the 2D-DIR-TSE images had a higher visual grade than the 3D-TSE images. Nonetheless, this difference was statistically insignificant (*P* = 0.065). Coronary artery points with a visual grade of 1 in either 3D-TSE or 2D-DIR-TSE images were removed from subsequent analyses.

**Table 2:** The summary of visual grading: Aneurysmal region, n = 27; Normal region, n = 21.

	Grade	Lumen boundary		Outer wall boundary	
		Aneurysm	Normal	Aneurysm	Normal
3D-TSE	4	1 (3.7%)	13 (61.9%)	3 (11.1%)	0 (0%)
	3	6 (22.2%)	7 (33.3%)	3 (11.1%)	9 (42.9%)
	2	15 (55.6%)	1 (4.8%)	9 (33.3%)	10 (47.6%)
	1	5 (18.5%)	0 (0%)	12 (44.4%)	2 (9.5%)
2D-DIR-TSE	4	0 (0%)	13 (61.9%)	3 (11.1%)	5 (23.8%)
	3	5 (18.5%)	7 (33.3%)	2 (7.4%)	6 (28.6%)
	2	15 (55.6%)	1 (4.8%)	12 (44.4%)	9 (42.9%)
	1	7 (25.9%)	0 (0%)	10 (37.0%)	1 (4.8%)
3D vs. 2D		*P* = 0.073	*P* = 1.000	*P* = 0.777	*P* = 0.065

*3D-TSE* three-dimensional turbo spin-echo, *2D-DIR-TSE* two-dimensional dual inversion-recovery turbo spin-echo.

Table 3 outlines the inter-reader and intra-reader reproducibility results. The inter- and intra-reader ICCs of all area measurements were > 0.8, thus indicating excellent reproducibility.

**Table 3:** The list of intraclass correlation coefficients between area measurement on inter- and intra-reader reproducibility, n = 34.

	LA		WA		NWI	
	3D-TSE	2D-DIR-TSE	3D-TSE	2D-DIR-TSE	3D-TSE	2D-DIR-TSE
Inter-reader	0.99 [0.97, 0.99]	1.00 [0.99, 1.00]	0.98 [0.95, 0.99]	0.98 [0.97, 0.99]	0.74 [0.50, 0.87]	0.80 [0.57, 0.91]
Intra-reader	0.99 [0.97, 0.99]	0.99 [0.98, 0.99]	0.95 [0.90, 0.98]	0.97 [0.93, 0.99]	0.73 [0.52, 0.86]	0.74 [0.49, 0.87]

[95% confidence interval].

*LA* lumen area, *WA* wall area, *NWI* normalized wall index.

**Figure 5:**
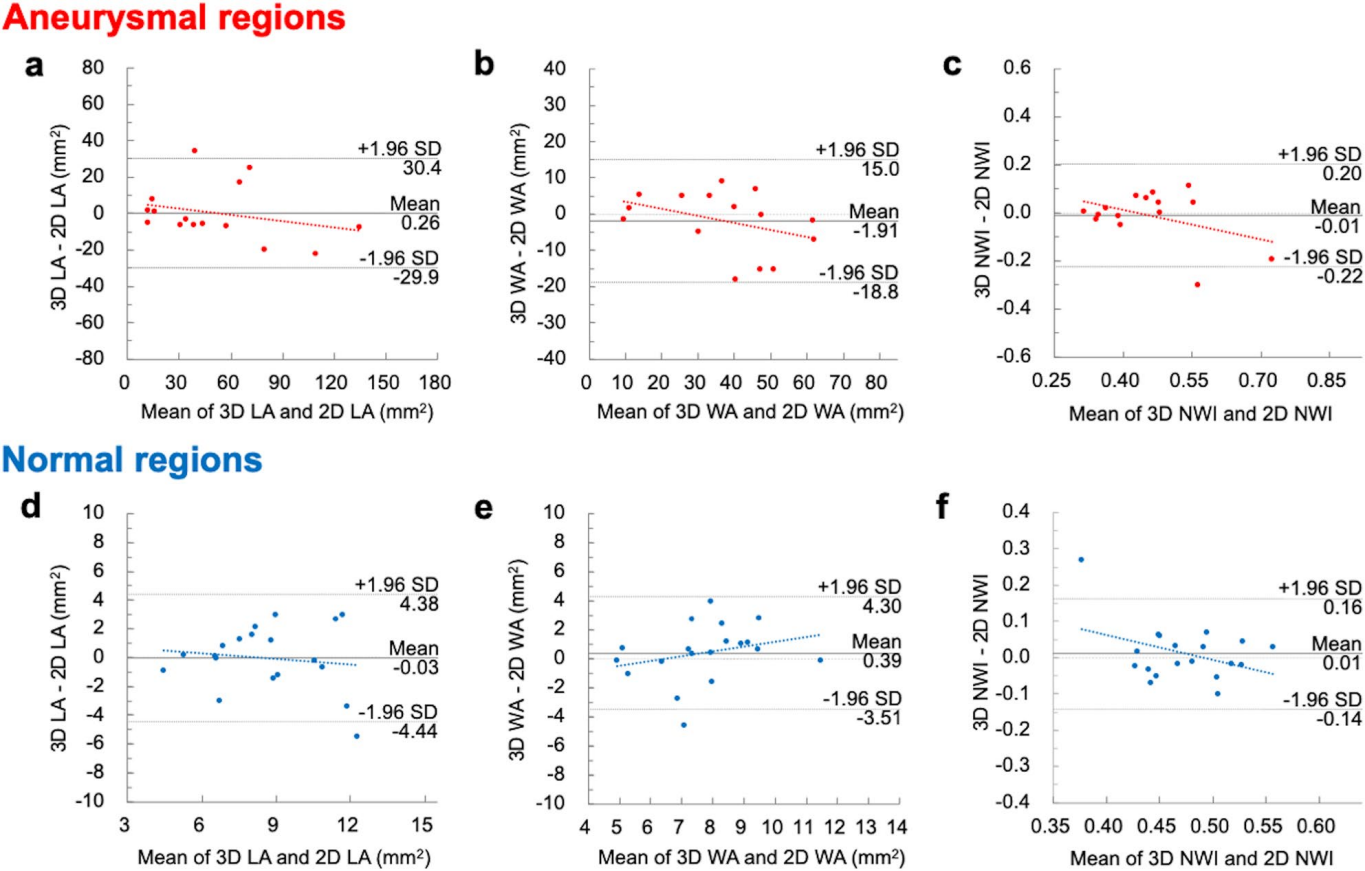
Bland–Altman plots of the LA, wall area WA, and NWI, determined by three-dimensional turbo spin-echo (3D-TSE) and 2D-DIR-TSE images in aneurysmal (**a–c**) and normal (**d–f**) regions. Bland–Altman plots demonstrate good agreement between 3D-TSE and 2D-DIR-TSE images. In the aneurysmal region, there are no fixed biases in LA, WA, and NWI (*P* = 0.948, 0.407, and 0.736, respectively). There are no proportional biases in LA, WA, and NWI (*P* = 0.334, 0.167, and 0.136, respectively). Similarly, in the normal regions, there are no fixed biases in LA, WA, and NWI (*P* = 0.956, 0.399, and 0.540, respectively). There are no proportional biases in LA, WA, and NWI (*P* = 0.600, 0.259, and 0.105, respectively).

**Figure 6:**
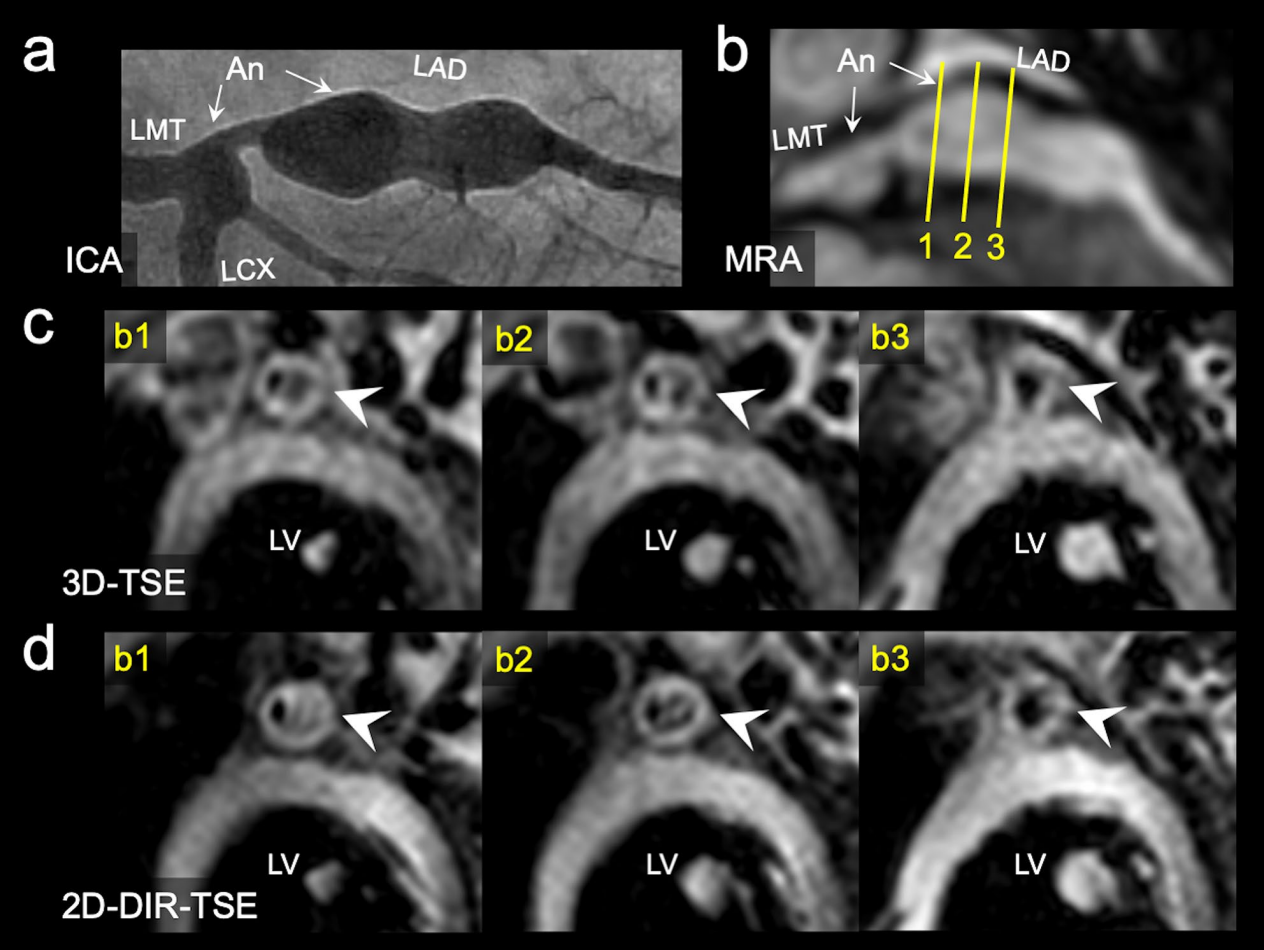
Invasive coronary angiography demonstrates two left coronary artery aneurysms (coronary artery segment 5, 4.5 mm; segment 6 to 7, 8.3 mm) in case 1 (**a**). In the MRA-collapsed partial MIP image, the yellow lines indicate aneurysmal regions (**b**). Each cross-sectional image of 3D-TSE and 2D-DIR-TSE corresponds to the solid line numbers in the MRA (**c,d**). In the 3D-TSE and 2D-DIR-TSE images, intraluminal signals in the aneurysm mimic thrombi because the flow signals are not suppressed. In the cross-section indicated by b2, the visual grading of the lumen boundary and outer wall boundary in 3D-TSE and 2D-DIR-TSE images have been grades 2 and 4, respectively, and depicted equally (arrowheads in (**c,d**)) (*An* aneurysm, *LMT* left main trunk, *LAD* left anterior descending artery, *LCX* left circumflex artery, *LV* left ventricle).

We observed high ICCs between 3D-TSE and 2D-DIR-TSE images in the LA and WA (LA, 0.95; WA, 0.95). Figure 5 depicts the Bland–Altman plots of the LA, WA, and NWI between the 3D-TSE and 2D-DIR-TSE images. The 95% limits of agreement were LA, WA, and NWI of − 29.9 to 30.4 mm^2^, − 18.8 to 15.0 mm^2^, and − 0.22 to 0.20 in the aneurysmal regions and − 4.44 to 4.38 mm^2^, − 3.51 to 4.30 mm^2^, and − 0.14 to 0.16 in the normal regions, respectively. There were no fixed and proportional biases between the 3D-TSE and 2D-DIR-TSE images in the aneurysmal and normal regions. Figure 6 depicts the representative 3D-TSE and 2D-DIR-TSE images.

## Discussion

We investigated the reproducibility of 3D-TSE and conventional 2D-DIR-TSE imaging in coronary vessel wall imaging in KD. To our knowledge, this is the first study to compare the 3D-TSE and 2D-DIR-TSE images in the coronary arteries. Qualitative and quantitative evaluations suggested reproducibility between the 3D-TSE and 2D-DIR-TSE images.

Despite slower flow velocity and larger influence of turbulence in the aneurysmal region than those in the normal region, the 3D-TSE images were equal to or better than the 2D-DIR-TSE images in the visual grade of the lumen boundary. High-resolution 3D-TSE imaging with variable flip angle trains (e.g., VISTA, SPACE, and CUBE) is advantageous for vessel wall imaging at various sites, considering its high scan efficiency, enabled by the use of long echo trains and its intrinsic black blood effect^15,20–23^. 3D-TSE imaging reportedly exerts a strong suppressive effect on blood flow signals in the coronary arteries. We used 3D-TSE imaging to select axial slice orientation. We observed suppression in coronary artery vessel wall imaging when the flow direction was almost parallel to the slice encoding direction. However, there was no suppression when the direction was almost perpendicular^11^. Aneurysms are often located in the proximal portion of the coronary artery^24^. By axially collecting the proximal portion of the coronary artery, the blood flow direction and the slice encoding direction become almost parallel, such that the blood flow signal was effectively suppressed.

In normal regions, the 3D-TSE images were equal to or less than the 2D-DIR-TSE images in the visual grade of the outer wall boundary. 3D-TSE imaging suggestively has a longer shot duration and scan time than 2D-DIR-TSE imaging, which increases the effects of heart action and respiration movement. In follow-up examination of KD, it is necessary not only to detect high-intensity thromboses but also to depict detailed anatomical structures, such as aneurysm regression. Considering the small anatomical size of coronary arteries and the movement during heart action and respiration, coronary vessel wall imaging remains more challenging than imaging of other vascular beds^25^. In a previous study, autopsy studies in subjects who did not die of coronary artery disease have revealed that the coronary vessel wall is typically 0.4–0.8 mm thick^26^. A previous report on the impact of spatial resolution on the accuracy of vessel WA measurement in simulations and phantom studies mentioned that a resolution ≤ 4 pixels across the wall leads to an overestimation of over 20%^27^. Moreover, coronary wall imaging reportedly requires a spatial resolution of 0.5 mm to 1 mm^28,29^. In the present study, the acquired spatial resolution of 1.17 × 1.25 × 1.40 mm was further reconstructed to 0.55 × 0.55 × 0.70 mm. Nonetheless, it is necessary to further improve spatial resolution with technological advancements in the future.

3D-TSE and 2D-DIR-TSE images had high ICCs and no bias, suggesting the images were reproducible. A previous study investigated the reproducibility of the internal diameter between 3D-TSE images and MRA^11^. The larger the diameter of the aneurysm, the narrower was the internal diameter of the 3D-TSE image than that of the MRA image. This may be attributed to laminar flow proximal to the vessel wall and increased turbulent flow caused by changes in the shape of the vessel lumen. In contrast, there was no bias in the internal diameter between 3D-TSE images and MRA in normal regions, thereby generating the expected range. The signal intensity of conventional 2D-DIR-TSE imaging relies on the inflow of fresh blood into the imaging slice between selective re-inversion and imaging. Therefore, aneurysmal regions are susceptible to flow direction and velocity. 2D-DIR-TSE images have a strong directivity in the acquisition section and flow direction, similar to 3D-TSE images.

The inter- and intra-reader reproducibility in the area measurement suggested that 3D-TSE images may be suitable for follow-up examination of the coronary vessel wall. 3D-TSE imaging also allows for easy visualization of accurate cross-sections, with less partial volume effects following postprocessing.

In recent years, computed tomographic coronary angiography (CTA) has been increasingly used due to decreasing radiation doses, high spatial resolution, and nearly isotropic data. CTA is less technically challenging than the MRI techniques used in this study. In Atherosclerosis, CTA can observe vulnerable plaques due to positive remodeling and low CT density^30^. Although, CTA is generally used to evaluate the vessel lumen rather than the vessel wall. On the other hand, MRI is a powerful technique that potentially allows the non-invasive investigation of the coronary vessel lumen, the coronary vessel wall, plaque morphology, and composition. Especially in childhood, non-invasive MRI, which does not require ionizing radiation and contrast agent, would allow serial investigation of disease.

To summarize the clinical advantages of 3D-TSE, the collection direction is the same axial collection as MRA, FOV settings are more convenient and practical because they were easier to determine and required less expertise. In the 3D-TSE, the aneurysm visualized by MRA can be visualized by reconstructing the data of the same cross-section or direction. Furthermore, 3D-TSE makes data more reproducible during follow-up MRI examination. Therefore, we suggest that 3D-TSE should be the first choice for coronary vessel wall imaging and 2D-DIR-TSE should be used as an option.

While this study generated significant findings, there were several limitations. First, we did not compare MR coronary vessel wall imaging with IVUS or OCT^11,31^, a standard approach for coronary artery wall assessments. However, IVUS is an invasive technique with non-negligible risks and is difficult to perform routinely. Second, the study was of very small size, and it is a single-center study with a single vendor. While 48 points were analyzed, a number of these were from the same coronary artery segments for each patient. The similarity among the results may be ruled out because flow direction changes at each cross-section of the aneurysmal regions. Although, there is a high possibility of a similarity in the normal regions. Finally, the voxel sizes of 3D-TSE and 2D-DIR-TSE imaging did not match. However, a trade-off occurs between high-resolution imaging and increased scan time, which can increase patient discomfort.

In conclusion, 3D-TSE imaging was reproducible with conventional 2D-DIR-TSE imaging for coronary vessel wall assessment of KD. The aneurysmal regions necessitate careful WA assessment, owing to the residual lumen signals of 3D-TSE and 2D-DIR-TSE images. 3D-TSE imaging may be suitable for follow-up examinations of KD because of its wide FOV, accurate cross-sectional images, and the reproducibility of area measurements.
